# Mitochondrial NADP^+^-dependent isocitrate dehydrogenase deficiency increases cisplatin-induced oxidative damage in the kidney tubule cells

**DOI:** 10.1038/s41419-018-0537-6

**Published:** 2018-04-25

**Authors:** Min Jung Kong, Sang Jun Han, Jee In Kim, Jeen-Woo Park, Kwon Moo Park

**Affiliations:** 1 Department of Anatomy, Cardiovascular Research Institute and BK21 Plus, School of Medicine, Kyungpook National University, 680 Gukchaebosang-ro, Junggu, Daegu, 41944 Republic of Korea; 2 Department of Molecular Medicine and MRC, Keimyung University School of Medicine, 1095 Dalgubeol-daero, Dalseogu, Daegu, 42601 Republic of Korea; 3 Department of Biochemistry, School of Life Sciences and Biotechnology, College of Natural Sciences, Kyungpook National University, Daegu, Republic of Korea

## Abstract

Mitochondrial NADP^+^-dependent isocitrate dehydrogenase (IDH2) plays an important role in the formation of NADPH, which is critical for the maintenance of mitochondrial redox balance. *Cis*-diamminedichloroplatinum II (cisplatin), an effective anticancer drug, induces oxidative stress-related nephrotoxicity, limiting its use. Therefore, we investigated whether IDH2, which is a critical enzyme in the NADPH-associated mitochondrial antioxidant system, is involved in cisplatin nephrotoxicity. *Idh2* gene-deleted (*Idh2*^−/−^) mice and wild-type (*Idh2*^*+/+*^) littermates were treated with cisplatin, with or without 2-(2,2,6,6-tetramethylpiperidin-1-oxyl-4-ylamino)-2-oxoethyl) triphenylphosphonium chloride (Mito-T), a mitochondria-specific antioxidant. Cisplatin-induced renal functional and morphological impairments were greater in *Idh2*^−/−^ mice than in *Idh2*^*+/+*^ mice. Mito-T mitigated those impairments in both *Idh2*^−/−^ and *Idh2*^*+/+*^ mice and this mitigation was greater in *Idh2*^−/−^ than in *Idh2*^*+/+*^ mice. Cisplatin impaired IDH2 function in the mitochondria, decreasing mitochondrial NADPH and GSH levels and increasing H_2_O_2_ generation; protein, lipid, and DNA oxidation; mitochondrial damage; and apoptosis. These cisplatin-induced changes were much more severe in *Idh2*^−/−^ mice than in *Idh2*^*+/+*^ mice. Mito-T treatment attenuated cisplatin-induced alterations in both *Idh2*^−/−^ and *Idh2*^*+/+*^ mice and this mitigation was greater in *Idh2*^−/−^ than in *Idh2*^*+/+*^ mice. Altogether, these data demonstrate that cisplatin induces the impairment of the mitochondrial IDH2-NADPH-GSH antioxidant system and IDH2 deficiency aggravates cisplatin-induced mitochondrial oxidative damage, inducing more severe nephrotoxicity. This suggests that the mitochondrial IDH2-NADPH-GSH antioxidant system is a target for the prevention of cisplatin-induced kidney cell death.

## Introduction

Cisplatin (cis-diamminedichloroplatinum II) is widely used as an effective chemotherapeutic reagent for malignant tumors. However, its nephrotoxicity limits its use^1,2^. This cisplatin nephrotoxicity, which causes acute kidney injury (AKI), is associated with oxidative stress of kidney tubular cells. Recent studies have demonstrated that cisplatin toxicity is highly associated with mitochondrial oxidative stress and subsequent mitochondrial dysfunction and cell death^3–5^. Cisplatin accumulates in the mitochondria of renal epithelial cells during excretion of cisplatin metabolites through renal tubules and further forms a cisplatin–glutathione (GSH) complex, which is easily released to the extracellular matrix through organic cation transporter 1 (OCT1) and copper transporter (Ctr1), consequently reducing mitochondrial GSH levels^6^. This causes the impairment of the GSH-associated mitochondrial antioxidant system, consequently increasing mitochondrial susceptibility to oxidative stress^3,6–8^.

Mitochondrial respiration produces reactive oxygen species (ROS), which can induce oxidative stress to cellular components, consequently leading to cell dysfunction and death. Therefore, mitochondria are well equipped with various antioxidant systems to cope with oxidative stress. However, pathological conditions, such as cisplatin nephrotoxicity, cause functional loss of mitochondrial antioxidant systems and overproduction of ROS, overwhelming their antioxidant capacity^9,10^. Mitochondrial oxidative stress eventually leads to mitochondrial dysfunction and damage, which can induce cell death^11–13^. In normal conditions, the superoxide anion produced in the mitochondria during mitochondrial respiration is primarily converted to toxic H_2_O_2_ by manganese superoxide dismutase (MnSOD). H_2_O_2_ is then further reduced to H_2_O by catalase, glutathione peroxidase (GSH-Px), and thiol-containing enzymes, such as thioredoxins (Trx), thioredoxin reductases (TrR), peroxiredoxins (Prx), and glutaredoxins^12,14,15^. GSH-Px is a family of tetrameric enzymes that contain the unique amino acid, selenocysteine, within their active sites and use low-molecular weight thiols, such as GSH, to reduce H_2_O_2_^14^. NADPH is commonly required for these antioxidants to provide a reducing equivalent, it maintains catalase in the active form, and it is used as a cofactor by TRX and glutathione reductase (GR), which convert oxidized GSH (GSSG) to GSH, a co-substrate for GSH-Px enzymes^14^. Therefore, NADPH is critical for the GSH-associated mitochondrial antioxidant system.

Intracellular NADPH is mainly generated by the reduction of NADP^+^ by glucose-6-phosphate dehydrogenase (G6PD) in the cytosol and NADP^+^-dependent isocitrate dehydrogenase 2 (IDH2) in the mitochondria^16^. Isocitrate dehydrogenases (IDHs) catalyze the oxidative decarboxylation of isocitrate to α-ketoglutarate, accompanied by the reduction of NAD(P)^+^ to NAD(P)H. Three IDHs, IDH1, IDH2, and IDH3, are present in mammals^17^. IDH1 and IDH2 are NADP^+^-dependent and they are localized in the cytosol and mitochondria, respectively^17^. IDH3 is NAD^+^-dependent and is localized in the mitochondria^17^. Recent evidence has demonstrated that NADPH levels generated by IDH1 and IDH2 are critical for the maintenance of redox balance via the GSH and thioredoxin systems of peroxide detoxification^18–21^.

Therefore, we hypothesized that IDH2 may be associated with cisplatin-induced AKI. In this study, we investigated the involvement of IDH2 in the mitochondrial NADPH-GSH antioxidant system in cisplatin nephrotoxicity using *Idh2* gene-deleted mice. Here, we report that cisplatin impairs the IDH2-NADPH-GSH-associated antioxidant system in the mitochondria, leading to mitochondrial oxidative stress and eventually, kidney tubular cell death and kidney dysfunction.

## Results

### IDH2 deficiency aggravates renal morphological and functional impairments after cisplatin administration

**Fig. 1:**
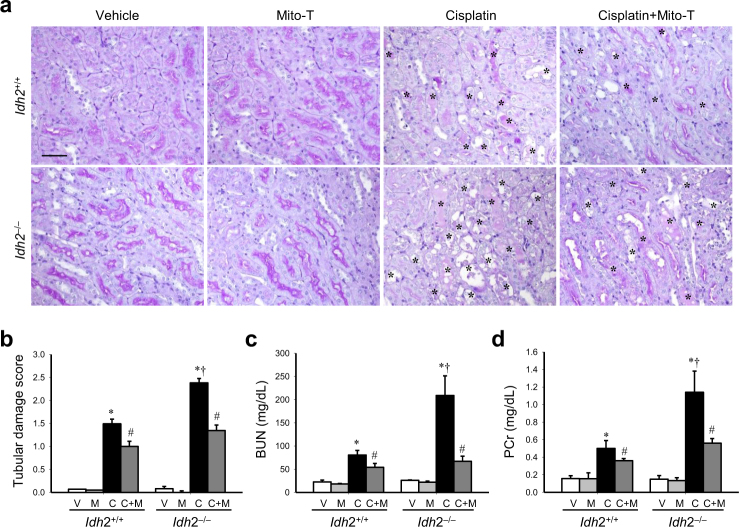
IDH2 deficiency aggravates renal histological and functional impairments after cisplatin administration. *Idh2*^*‒/‒*^ mice and wild-type (*Idh2*^+/+^) littermates were intraperitoneally injected with either cisplatin (C, 20 mg/kg B.W.) or 0.9% saline (vehicle, V) once. Some mice were treated with Mito-T (M, 0.7 mg/kg B.W.) daily, beginning 7 days before cisplatin injection and continuing until experiments were completed. Three days after cisplatin injection, renal functional and histological impairment were determined. **a** Kidney sections were stained with periodic acid Schiff reagent. Asterisks indicate damaged tubules. **b** Tubular damage score was obtained as described in the “Materials and methods” section. **c**, **d** Concentrations of blood urea nitrogen (BUN) and plasma creatinine (PCr) were determined 3 days after cisplatin injection. Results are expressed as means ± SE (*n* = 5). Scale bars: **a** 100 µm. *p < 0.05 vs. respective V; ^#^*p* < 0.05 vs. respective C; ^§^*p* < 0.05 vs. V in *Idh2*^+/+^; ^†^*p* < 0.05 vs. C in *Idh2*^+/+^

To investigate whether IDH2 deletion affects cisplatin-induced AKI, we evaluated kidney morphology and function. Cisplatin induced the loss of the brush border in tubular epithelial cells and dilation and congestion of tubules in both *Idh2*^+/+^ and *Idh2*^*‒/‒*^ mice (Fig. 1a, b). Tubular cell damage was greatest in the proximal tubular cells compared with other tubular cells (Fig. 1a). Consistent with tubular damage, blood urea nitrogen (BUN) and plasma creatinine (PCr) levels were markedly increased in the cisplatin-treated mice (Fig. 1c, d). These morphological and functional effects in the kidney after cisplatin injection were higher in *Idh2*^*‒/‒*^ mice than in wild-type littermates (Fig. 1). Mito-T, a mitochondria-targeting antioxidant molecule, ameliorated this cisplatin-induced histological and functional damage in both *Idh2*^+/+^ and *Idh2*^*‒/‒*^ mice and this amelioration was more profound in *Idh2*^*‒/‒*^ mice than in *Idh2*^+/+^ mice (Fig. 1); Mito-T treatment reduced tubular damage score by ~45% and 27% in cisplatin-injected *Idh2*^*‒/‒*^ and *Idh2*^+/+^ mice, respectively (Fig. 1b). The decreases in BUN after Mito-T treatment were ~68% and 30% in *Idh2*^*‒/‒*^ and *Idh2*^+/+^ mice, respectively (Fig. 1c). Reductions of PCr after Mito-T treatment were ~51% and 28% in *Idh2*^*‒/‒*^ and *Idh2*^+/+^ mice, respectively (Fig. 1d). These results indicate that IDH2 deficiency elevates cisplatin nephrotoxicity.

### IDH2 deficiency augments mitochondrial oxidative stress after cisplatin administration

**Fig. 2:**
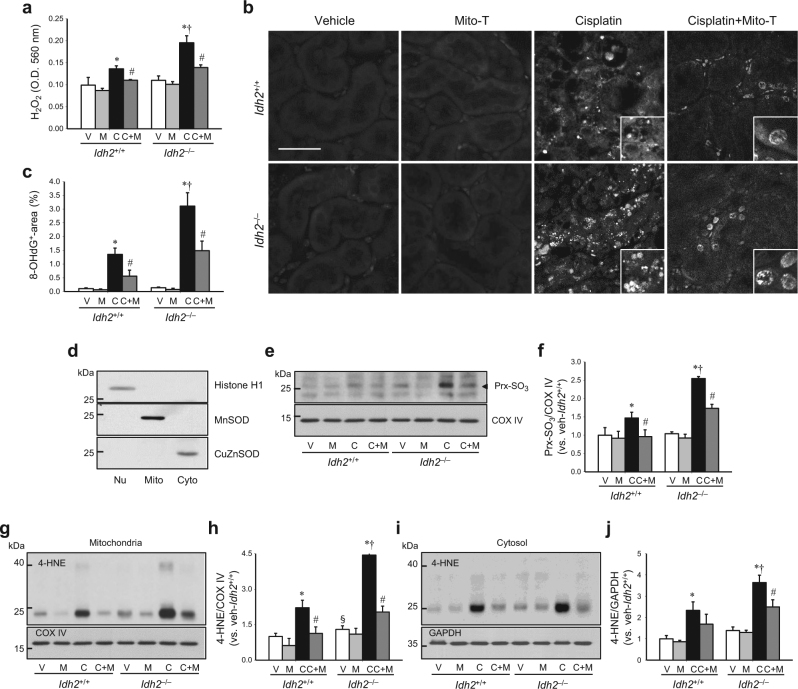
IDH2 deficiency exacerbates hydrogen peroxide formation and oxidation of DNA in the kidney after cisplatin administration. *Idh2*^*‒/‒*^ mice and wild-type (*Idh2*^+*/+*^) littermates were intraperitoneally injected with either cisplatin (C, 20 mg/kg B.W.) or 0.9% saline (vehicle, V) once. Some mice were treated with Mito-T (M, 0.7 mg/kg B.W.) daily, beginning 7 days before cisplatin injection and continuing until experiments were completed. Kidneys were harvested 3 days after cisplatin injection. **a** H_2_O_2_ was measured in whole kidney tissue (*n* = 4-5). **b** Kidney sections were immunostained with an anti-8-hydroxy-2′-deoxyguanosine (8-OHdG) antibody. **c** 8-OHdG-positive area (%) was measured. More than 10 fields per kidney section were analyzed (*n* = 3). **d** Fractions were confirmed by western blot analysis using anti-MnSOD for the mitochondria, -CuZnSOD for the cytosol, and -histone H1 for the nucleus. **e**, **h** Expression levels of Prx-SO_3_ and 4-hydroxynonenal (4-HNE) were determined in the mitochondrial fraction of kidneys by western blot analysis. **f**, **h** Band densities were normalized to COX IV band densities using the ImageJ program. COX IV bands in “**e**” and “**g**” are same band produced by same blot. **i** Expression of 4-HNE was detected in cytosolic fraction of kidneys by western blot analysis. GADPH was used as a loading control. **j** Band density was normalized to GAPDH band density using the ImageJ program. Results are expressed as means ± SE (*n* = 3–5 per group). Scale bars: **b** 50 µm. *p < 0.05 vs. respective V; ^#^*p* < 0.05 vs. respective C; ^§^*p* < 0.05 vs. V in *Idh2*^+/+^; ^†^*p* < 0.05 vs. C in *Idh2*^+/+^. Nu nucleus, Mito mitochondria, Cyto cytosol

To investigate whether high susceptibility to cisplatin in *Idh2*-gene-deficient mice is associated with oxidative stress, we first determined ROS formation in the kidney. Cisplatin injection increased H_2_O_2_ production and 8-OHdG, an index of oxidized DNA, signals in both *Idh2*^+/+^ and *Idh2*^*‒/‒*^ mouse kidneys and these cisplatin-induced increases were higher in *Idh2*^*‒/‒*^ mice than in *Idh2*^+/+^ mice (Fig. 2a–c). The 8-OHdG antibody binds to DNA damaged by oxidation in mitochondria and nuclei^22^. Therefore, these increased 8-OHdG signals indicate increased nuclear and mitochondrial DNA oxidation. Next, we measured the mitochondrial oxidative stress by western blot analysis using anti-Prx-SO_3_, an oxidized form of Prx, and -4-hydroxynoneal (4-HNE), an oxidized lipid, antibody in the mitochondria. Mitochondrial and cytosolic fraction was confirmed through western blot analysis using anti-manganese superoxide dismutase (MnSOD) for the mitochondria, -copper-zinc superoxide dismutase (CuZnSOD) for the cytosol, and -histone H1 for the nucleus antibodies, respectively (Fig. 2d). Mitochondrial Prx-SO_3_ and 4-HNE expression also increased in cisplatin-injected mice and these increases were greater in *Idh2*^*‒/‒*^ mice than in *Idh2*^+/+^ mice (Fig. 2e–h). In addition, we determined 4-HNE expression in cytosol, since mitochondrial oxidative stress can extend into the cytosol, and vice versa. 4-HNE expression in the cytosol also increased in cisplatin-injected mice and this increase was greater in *Idh2*^*‒/‒*^ mice than in *Idh2*^+/+^ mice (Fig. 2i, j). These cisplatin-induced increases in H_2_O_2_, 8-OHdG, Prx-SO_3_, and 4-HNE were significantly attenuated by Mito-T treatment in both *Idh2*^*‒/‒*^ and *Idh2*^+/+^ mouse kidneys (Fig. 2). Attenuation by Mito-T was greater in *Idh2*^*‒/*‒^ mice than in *Idh2*^+/+^ mice (Fig. 2). Taken together, these results indicate that cisplatin induces mitochondrial oxidative stress and IDH2 deficiency augments cisplatin-induced mitochondrial oxidative injury. Therefore, increased susceptibility caused by *Idh2* gene deletion may be associated with the increased mitochondrial damage.

### Cisplatin impairs the IDH2-NADPH-GSH-associated mitochondrial antioxidant system and *Idh2* gene deletion exacerbates this cisplatin-induced impairment

**Fig. 3:**
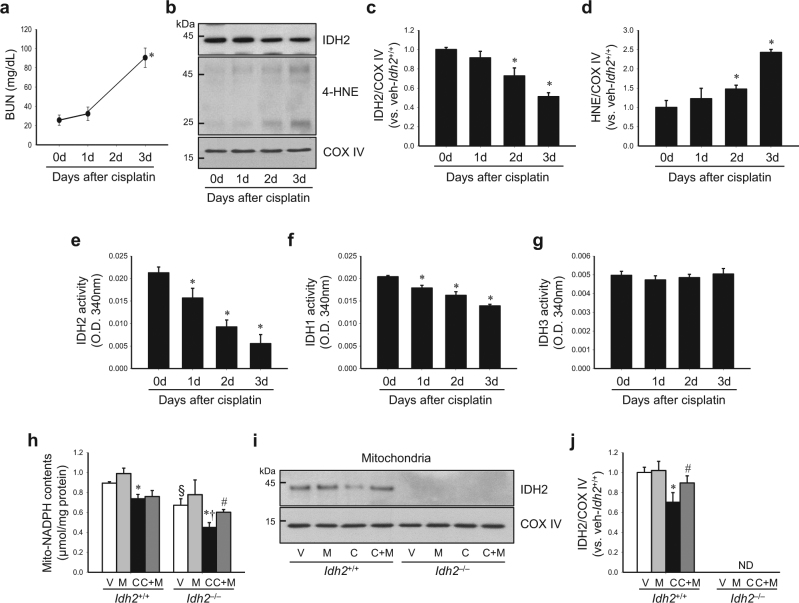
Cisplatin administration decreases IDH2 expression and NADPH levels. *Idh2*^*‒/‒*^ mice (**h**–**j**) and wild-type (*Idh2*^+/+^) littermates (**a**–**j**) were intraperitoneally injected with either cisplatin (C, 20 mg/kg B.W.) or 0.9% saline (vehicle, V) once. Some mice were treated with Mito-T (M, 0.7 mg/kg B.W.) daily, beginning 7 days before cisplatin injection and continuing until experiments were completed. Kidneys were harvested at the indicated time after cisplatin injection. **a** BUN concentration was determined at the indicated time (*n* = 4). **b** IDH2 and 4-HNE expression in mitochondrial fraction was determined by western blot analysis. **c**, **d** Band densities were measured by the ImageJ program. COX IV was used as loading control (*n* = 4). **e**–**g** Activities of IDH2 (**e**), IDH1 (**f**), and IDH3 (**g**) were measured in mitochondria fraction (**e**, **g**) and cytosol fraction (**f**), respectively (*n* = 4). **h** NADPH concentration in the kidney mitochondrial fraction was measured as described in the “Materials and methods” section. **i** Expression of IDH2 in mitochondrial fraction was detected by western blot analysis. **j** Band density was normalized to COX IV band using the ImageJ program. Results are expressed as means ± SE (*n* = 3–4 per group). *p < 0.05 vs. respective V; ^#^*p* < 0.05 vs. respective C; ^§^*p* < 0.05 vs. V in *Idh2*^+/+^; ^†^*p* < 0.05 vs. C in *Idh2*^+/+^. ND non-detectable

First, we determined whether cisplatin affects IDH2 function and expression. Cisplatin injection greatly decreased the activity of IDH2 in the mitochondria from 1 day after injection with mild or no increase in the expression level (Fig. 3b, c, e), even when increases of BUN and 4-HNE expression were not observed, or very mild (Fig. 3a, b, d). The decrease of IDH2 activity was exacerbated overtime in a time-dependent manner (Fig. 3e). IDH2 expression also decreased after cisplatin injection in the same pattern as activity (Fig. 3b, c). Cisplatin also decreased IDH1 activity in cytosol. This decrease in IDH1 activity was less when compared with the decrease of IDH2 activity (Fig. 3f). However, IDH3 activity in the mitochondria was not significantly changed after cisplatin injection (Fig. 3g). Although these results cannot provide an answer regarding causality, the above data suggest that the decrease of IDH2 function through cisplatin could be a cause at least in part.

Next, we determined whether cisplatin decreased NADPH levels in the mitochondria in both *Idh2*^+/+^ mice and *Idh2*^−/−^ mice (Fig. 3h). Compared to *Idh2*^+/+^ mice, *Idh2*^−/−^ mice showed a greater decrease in NADPH level after cisplatin injection (Fig. 3h). NADPH levels in the mitochondrial fraction of vehicle-treated *Idh2*^*‒/‒*^ mouse kidneys were lower than that in vehicle-treated *Idh2*^+/+^ mice (Fig. 3h). In addition, cisplatin reduced IDH2 expression and this reduction was prevented by Mito-T treatment (Fig. 3i, j). These results indicate that IDH2 regulates NADPH level in the mitochondria and cisplatin impairs the NADPH-producing system of mitochondria.

**Fig. 4:**
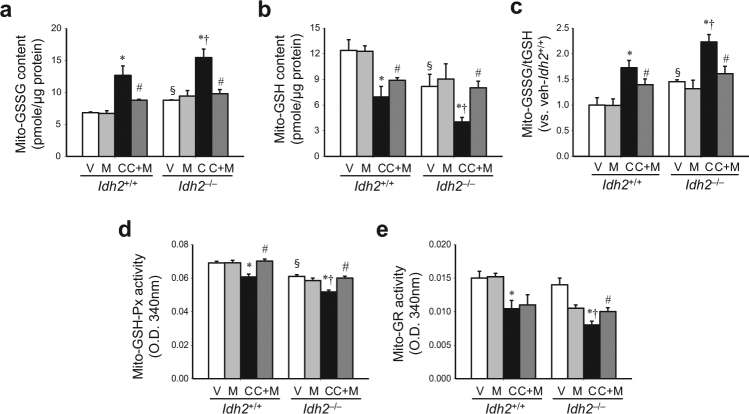
Cisplatin administration impairs the mitochondrial GSH-mediated antioxidant system in the kidney. *Idh2*^*‒/‒*^ mice and wild-type (*Idh2*^+/+^) littermates were intraperitoneally injected with either cisplatin (C, 20 mg/kg B.W.) or 0.9% saline (vehicle, V) once. Some mice were treated with Mito-T (M, 0.7 mg/kg B.W.) daily, beginning 7 days before cisplatin injection and continuing until experiments were completed. Kidneys were harvested 3 days after cisplatin injection. **a**–**c** Oxidized GSH (GSSG) levels (**a**), reduced GSH levels (**b**), and the GSSG/tGSH ratio (**c**) were determined in mitochondrial fractions. **d**, **e** GSH-Px (**d**) and GR (**e**) activities were measured in mitochondrial fractions. Results are expressed as means ± SE (*n* = 4 per group). *p < 0.05 vs. respective V; ^#^*p* < 0.05 vs. respective C; ^§^*p* < 0.05 vs. V in *Idh2*^+/+^; ^†^*p* < 0.05 vs. C in *Idh2*^+/+^

Further, we determined the ratio of oxidized glutathione (GSSG) to total glutathione (tGSH) in the mitochondrial fraction of kidneys, because NADPH plays a critical role in the reduction of GSSG to GSH. After cisplatin treatment, mitochondrial GSSG levels increased in both *Idh2*^+/+^ and *Idh2*^*‒/‒*^ mouse kidneys, whereas GSH levels decreased (Fig. 4a, b). These changes increased the GSSG/tGSH ratio in both *Idh2*^+/+^ and *Idh2*^*‒/‒*^ mice, showing a higher increase in *Idh2*^*‒/‒*^ mouse kidneys than in *Idh2*^+/+^ mouse kidneys (Fig. 4c). Mito-T significantly attenuated the cisplatin-induced changes in GSSG and GSH in both mice. However, these mitigations after Mito-T treatment were more prominent in *Idh2*^*‒/‒*^ mice compared with their wild-type littermates (Fig. 4a–e).

Finally, we determined the activity of GSH-Px, which uses GSH as a substrate, and GR, which reduces GSSG to GSH using NADPH^12^. Mitochondrial GSH-Px and GR activities decreased in both *Idh2*^+/+^ and *Idh2*^*‒/‒*^ mice after cisplatin injection (Fig. 4d, e). These reduced activities were greater in *Idh2*^*‒/‒*^ mice than in *Idh2*^+/+^ mice (Fig. 4d, e). The reduction in GSH-Px and GR activities was significantly attenuated by Mito-T and this attenuation was higher in *Idh2*^*‒/‒*^ than in *Idh2*^+/+^ mice (Fig. 4d, e). These data indicate that cisplatin impairs the mitochondrial NADPH-GSH antioxidant system.

### IDH2 deficiency accelerates mitochondrial damage following cisplatin administration

**Fig. 5:**
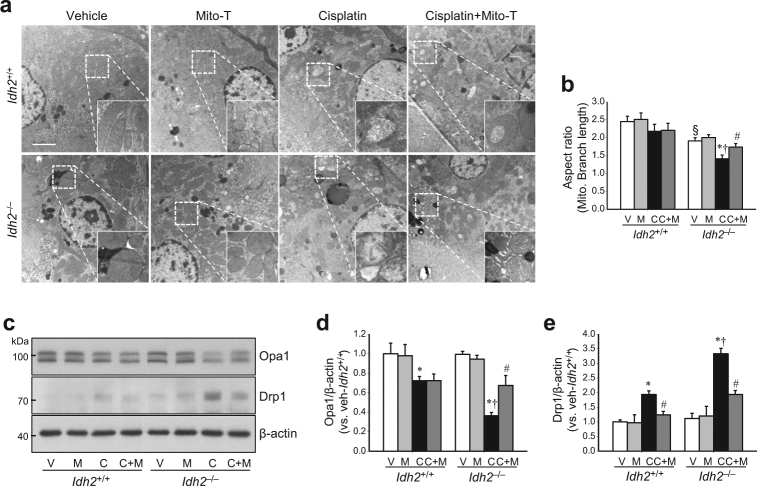
IDH2 deficiency augments mitochondrial damage after cisplatin administration. *Idh2*^*‒/‒*^ mice and wild-type (*Idh2*^+/+^) littermates were intraperitoneally injected with either cisplatin (C, 20 mg/kg B.W.) or 0.9% saline (vehicle, V) once. Some mice were treated with Mito-T (M, 0.7 mg/kg B.W.) daily, beginning 7 days before cisplatin injection and continuing until experiments were completed. **a** Two days after cisplatin administration, mitochondrial structures were examined by transmission electron microscopy (TEM). Higher magnification is shown by the dash-lined rectangles. Scale bar indicates 2 μm. **b** The mitochondrial aspect ratio [(major axis)/(minor axis)] was computed using 30 mitochondria per cell. **c** Expressions of OPa1 and Drp1 were determined by western blot analysis. β-actin was used as a loading control. **d**, **e** OPa1 (**d**) and Drp1 (**e**) band densities were measured using the ImageJ program. Results are expressed as means ± SE (*n* = 3–4 per group). Scale bars: **a** 2 µm. *p < 0.05 vs. respective V; ^#^*p* < 0.05 vs. respective C; ^§^*p* < 0.05 vs. V in *Idh2*^+/+^; ^†^*p* < 0.05 vs. C in *Idh2*^+/+^

Oxidative stress in mitochondria induces mitochondrial dysfunction and a shift of the mitochondrial dynamics toward fission, leading to mitochondrial fragmentation and activation of the apoptotic signal pathway^23,24^. Therefore, we investigated whether increased susceptibility to cisplatin in *Idh2*^*‒/‒*^ mice is associated with mitochondrial damage. Cisplatin induced damage of mitochondria in the proximal tubular cells of both *Idh2*^+/+^ and *Idh2*^*‒/‒*^ mouse kidneys (Fig. 5a). This mitochondrial damage was more severe in *Idh2*^*‒/‒*^ mice than in *Idh2*^+/+^ mice together with higher mitochondrial aspect ratio [(major axis/minor axis)] in the *Idh2*^*‒/‒*^ mice than *Idh2*^+/+^ mice (Fig. 5a, b). Because mitochondrial fragmentation is associated with regulatory proteins of mitochondrial fusion and fission^9^, we determined the expression of mitochondrial fusion and fission regulatory proteins. Cisplatin decreased the expression of Opa1, a regulator of mitochondrial fusion, in both *Idh2*^+/+^ and *Idh2*^*‒/‒*^ mouse kidneys (Fig. 5c, d). Conversely, Drp1, a mitochondria fission protein, was increased by cisplatin administration (Fig. 5c, e). These cisplatin-induced alterations in Opa1 and Drp1 expression were greater in *Idh2*^*‒/‒*^ than in *Idh2*^+/+^ mice (Fig. 5c–e). Mito-T treatment preserved mitochondrial morphology (Fig. 5a) and significantly mitigated the changes in Opa1 and Drp1 expression in both *Idh2*^+/+^ and *Idh2*^*‒/‒*^ mice (Fig. 5c–e). These Mito-T effects were more dramatic in *Idh*2^*‒/‒*^ mice than in their wild-type littermates (Fig. 5c–e). These results indicate that *Idh*2 gene deletion exacerbates cisplatin-induced mitochondrial damage.

### IDH2 deficiency augments apoptosis after cisplatin administration

**Fig. 6:**
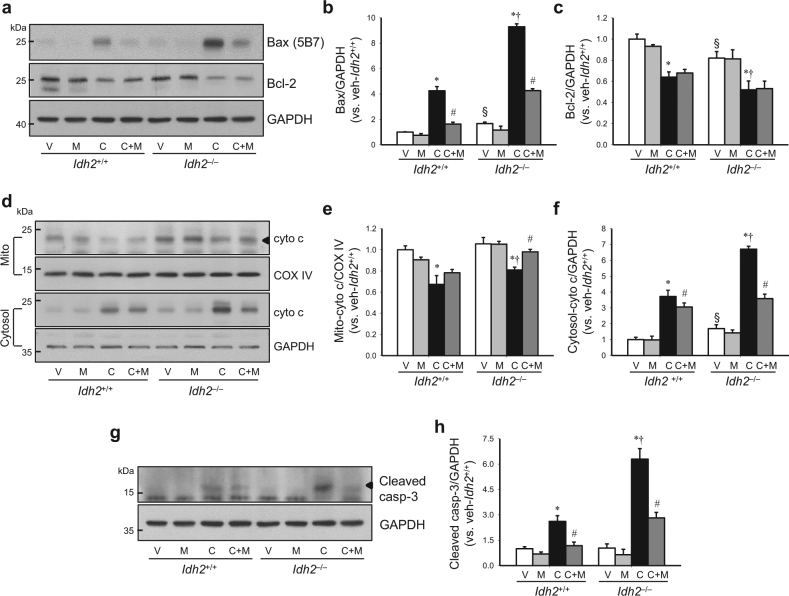
IDH2 deficiency exacerbates apoptosis after cisplatin administration. *Idh2*^*‒/‒*^ mice and wild-type (*Idh2*^+/+^) littermates were intraperitoneally injected with either cisplatin (C, 20 mg/kg B.W.) or 0.9% saline (vehicle, V) once. Some mice were treated with Mito-T (M, 0.7 mg/kg B.W.) daily, beginning 7 days before cisplatin injection and continuing until experiments were completed. Kidneys were harvested 3 days after cisplatin injection. **a**–**c** Bax and Bcl-2 expression in whole kidney lysates were determined by western blot analysis. GAPDH was used as a loading control. **b**, **c** Bax (**b**) and Bcl-2 (**c**) band densities were measured using the ImageJ program. **d**–**f** Mitochondria and cytosol were fractioned as described in the “Materials and methods” section. Cytochrome *c* expression was determined in mitochondrial and cytosolic fractions by western blot analysis. **e**, **f** Band densities were measured using the ImageJ program and normalized to COX IV and GAPDH. **g** Cleaved caspase-3 was detected in whole kidney lysates by western blot analysis. **h** Band density was measured using the ImageJ program and normalized to GAPDH. Results are expressed as means ± SE (*n* = 3–5 per group). *p < 0.05 vs. respective V; ^#^*p* < 0.05 vs. respective C; ^§^*p* < 0.05 vs. V in *Idh2*^+/+^; ^†^*p* < 0.05 vs. C in *Idh2*^+/+^

Because mitochondrial oxidative stress activates apoptosis signaling pathways^13,25^, we examined whether *Idh2* gene deletion affects apoptosis after cisplatin injection. First, we investigated the responses of the apoptosis regulatory signal pathway after cisplatin injection in both *Idh2*^+/+^ and *Idh2*^‒/‒^ mouse kidneys. Cisplatin increased Bax expression, whereas it decreased Bcl-2 expression in both *Idh2*^+/+^ and *Idh2*^*‒/‒*^ mouse kidneys. Changes in expression were greater in *Idh2*^*‒/‒*^ than in *Idh2*^+/+^ mice (Fig. 6a–c). Mito-T treatment significantly inhibited the cisplatin-induced increase in Bax, but not Bcl-2 expression (Fig. 6a–c). Cisplatin induced the release of cytochrome *c* from mitochondria into the cytosol in both *Idh2*^+/+^ and *Idh2*^*‒/‒*^ mice, and this release was greater in *Idh2*^*‒/‒*^ mice than in *Idh2*^+/+^ mice (Fig. 6d–f). Cleaved caspase-3 expression levels were also elevated by cisplatin injection in both *Idh2*^+/+^ and *Idh2*^*‒/‒*^ mice, and this elevation was greater in *Idh2*^*‒/‒*^ mice than in *Idh2*^+/+^ mice (Fig. 6g, h). Mito-T reduced cytochrome *c* release and cleavage of caspase-3 in both *Idh2*^*‒/‒*^ and *Idh2*^+/+^ mice, showing a greater reduction in *Idh2*^*‒/‒*^ mice than in *Idh2*^+/+^ mice (Fig. 6d–h).

**Fig. 7:**
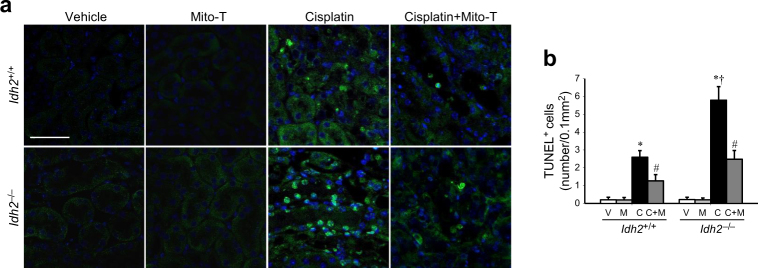
IDH2 deficiency exacerbates apoptosis after cisplatin administration. *Idh2*^*‒/‒*^ mice and wild-type (*Idh2*^+/+^) littermates were intraperitoneally injected with either cisplatin (C, 20 mg/kg B.W.) or 0.9% saline (vehicle, V) once. Some mice were treated with Mito-T (M, 0.7 mg/kg B.W.) daily, beginning 7 days before cisplatin injection and continuing until experiments were completed. Kidneys were harvested 3 days after cisplatin injection. Kidney sections were stained using a TUNEL assay kit. **a** Immunostaining shows TUNEL-positive cells (green). Nuclei were stained with DAPI (blue). **b** TUNEL-positive cells were counted in 10 fields per kidney. Results are expressed as means ± SE (*n* = 3 per group). Scale bars: **a** 50 µm. *p < 0.05 vs. respective V; ^#^*p* < 0.05 vs. respective C; ^†^*p* < 0.05 vs. C in *Idh2*^+/+^

Finally, we determined the apoptosis of kidney tubular cells by terminal deoxynucleotidyl transferase dUTP nick end labeling (TUNEL) analysis. Cisplatin injection increased the number of TUNEL-positive tubular epithelial cells (Fig. 7a). This increase in TUNEL-positive cells was greater in *Idh2*^*‒/‒*^ mice than in their wild-type littermates (Fig. 7a, b). Mito-T reduced the cisplatin-induced increase in TUNEL-positive cells in both *Idh2*^*‒/‒*^ and *Idh2*^+/+^ mice (Fig. 7a, b). This effect of Mito-T was greater in *Idh2*^*‒/‒*^ mice than in *Idh2*^+/+^ mice (Fig. 7a, b). These results indicate that IDH2 deficiency exacerbates cisplatin-induced apoptosis, which is associated with mitochondrial oxidative stress.

## Discussion

In the present study, we have reported, for the first time, that cisplatin impairs the mitochondrial IDH2-NADPH-GSH antioxidant system, leading to mitochondrial oxidative stress and consequently, renal cell death and dysfunction. In addition, *Idh2* gene deletion reduces mitochondrial NADPH levels and exacerbates cisplatin-induced mitochondrial oxidative stress and kidney injury. Furthermore, the mitochondria-targeting antioxidant, Mito-T, mitigates cisplatin nephrotoxicity. These findings suggest that IDH2 is associated with cisplatin-induced nephrotoxicity and the IDH2-NADPH-GSH axis considered as a target pathway to develop preventive treatments for cisplatin nephrotoxicity and AKI.

When cisplatin is excreted through urination, cisplatin accumulates in kidney cells by uptake through OCT1 and Ctr1, which are highly expressed on kidney tubular epithelial cells^6,7^. Cisplatin metabolites have a positive charge by hydration and therefore, easily penetrate the mitochondrial membrane, because the mitochondria has a negative membrane potential (~180 mV). The metabolites then accumulate in the mitochondria and combine with negatively charged mitochondrial components, such as DNA, RNA, and proteins, leading to loss of function, including their antioxidant functions^26,27^. Therefore, mitochondria-rich cells, such as proximal tubular cells, are very susceptible to cisplatin^25^. In the present study, we found that proximal tubular cell damage is most severe among kidney tubular cells, suggesting that cisplatin nephrotoxicity is associated with mitochondrial density and damage. Because IDH2 is abundant in the proximal tubular cells^16^, *Idh2*-gene-deleted mice may be more susceptible to cisplatin toxicity in these cells.

Cisplatin induces ROS formation by inhibiting complexes I–IV of the respiratory chain and inhibiting GR function, which leads to decreased GSH levels, consequently resulting in increased mitochondrial ROS and oxidative stress, which can expand to entire cells^28^. Many studies have demonstrated that the reduction of mitochondrial ROS attenuates renal injury during cisplatin administration^11,29,30^. On the contrary, weakening of antioxidant capacity, such as by GSH deletion, exacerbates cisplatin nephrotoxicity^30^. Furthermore, cisplatin decreased mitochondrial GSH levels and GSH-Px and GR activity, leading to increases in mitochondrial H_2_O_2_ levels; oxidation of DNA, proteins, and lipids; mitochondrial swelling; cristae loss; and a shift to fission in proximal tubular cells. These cisplatin-induced changes were enhanced by *Idh2* gene deletion, whereas a mitochondria-targeting antioxidant inhibited these cisplatin-induced changes in both *Idh2*^*‒/‒*^ and *Idh2*^+/+^ mice, but with a greater effect in *Idh2*^*‒/‒*^ mice. In addition, in this present study we found that the activity of IDH2 declined from 1 day after cisplatin injection, when renal functional impairment and oxidative stress are very mild or absent. This result indicates that cisplatin impairs IDH2 function, suggesting that the decline of IDH2 activity is not only a secondary response to oxidative stress, but also a primary response in cisplatin-nephrotoxicity. However, to define how cisplatin impairs IDH2 function, further studies are required.

NADPH is essential for the GSH-associated mitochondrial antioxidant system by providing a reducing equivalent, maintaining catalase in the active form, and acting as a cofactor for Trx and GR, which convert GSSG to GSH, a substrate for the GSH-Px enzymes^14^. NADPH does not shuttle between the mitochondria and the cytosol^14,31^. We found that IDH1 activity declined after cisplatin injection, although the decline was relatively milder than the decline of IDH2 activity. This suggests that, although cisplatin toxicity is associated with both IDH1 and IDH2, IDH2 plays more critical role in cisplatin nephrotoxicity. We speculate that the smaller decline in IDH1 may be due to the functions of enzymes that produce NADPH. NADPH in the cytosol is mainly produced by G6PD^19^. However, mitochondrial oxidative stress extends into the cytosol, and vice versa, hence IDH1 can be also associated with cisplatin-induced nephrotoxicity. Since IDH3 is a family member of IDH, although IDH3 is not critically involved in the production of NADPH^19^, we determined IDH3 activity. As expected, we did not find any significant change in IDH3 activity in the kidneys after cisplatin injection. Therefore, these data indicate that mitochondrial NADPH production by IDH2 is critical for the maintenance of mitochondrial redox balance. In the present study, we found that *Idh2* gene deletion decreased NADPH levels in kidney. The *Idh2* gene deletion also decreased GSH levels in the mitochondria of kidney cells. Furthermore, *Idh2* gene deletion augmented the cisplatin-induced increase in the GSSG/tGSH ratio and decreased GSH-Px and GR activities in the mitochondrial fraction of kidneys. These data indicate that IDH2 regulates mitochondrial NADPH levels and mitochondrial GSH-associated antioxidant systems, suggesting that IDH2 is involved in cisplatin-induced nephrotoxicity via affecting mitochondrial NADPH levels.

Increased oxidative stress causes mitochondrial fragmentation and enhances the release of mitochondria apoptotic factors, leading to cell death^9,23,32,33^. In the present study, cisplatin induced severe mitochondrial injury and dysfunction and disturbed the balance of mitochondrial fission and fusion protein expression, resulting in a shift of mitochondria to fission, which then increased mitochondria fragmentation. *Idh2* gene deletion promoted this cisplatin-induced mitochondrial damage. Furthermore, disruption of mitochondria leads to translocation of the pro-apoptotic factor, Bax, from the cytosol to mitochondria and degradation of anti-apoptotic proteins, such as Bcl-2, leading to apoptosis and/or necrosis^4,9,32^. Wang et el. reported that Drp1, a mitochondrial fission protein, activates Bax translocation from the cytosol to mitochondria, leading to the formation of a mitochondrial membrane pore, consequently resulting in the release of cytochrome *c* to the cytosol^34^. In the present study, cisplatin induced the release of cytochrome *c* from mitochondria to the cytosol, with increases in Drp1 and Bax expression. These cisplatin-induced alterations were greater in *Idh2*^*‒/‒*^ mice than in wild-type mice. These data indicate that increased mitochondrial oxidative stress in *Idh2*^*‒/‒*^ mice is associated with mitochondrial fragmentation and activation of apoptosis signaling pathways. It has been reported that, when ROS formation overwhelms mitochondrial antioxidant capacity, apoptosis is triggered^16,35^. ROS production above mitochondrial antioxidant capacity triggers the opening of the mitochondrial permeability transition (MPT) pore and induces the release of cytochrome *c* from mitochondria to the cytosol, activating the mitochondria-dependent intrinsic apoptosis pathway and leading to cell death^33,35,36^. There is evidence that inhibition of mitochondrial ROS and reduction of mitochondrial oxidative stress reduces cisplatin toxicity^11–13^. In the present study, Mito-T, a mitochondria-targeting antioxidant molecule, prevented cisplatin-induced changes in Bax and Bcl-2 expression and cytochrome *c* release, resulting in reduced apoptosis of kidney tubular epithelial cells. These changes were higher in *Idh2*^*‒/‒*^ mice than in *Idh2*^+/+^ mice. In addition, Mito-T treatment inhibited the increase in Drp1 expression. Furthermore, the protective effect of Mito-T was greater in *Idh2*^*‒/‒*^ mice compared with their wild-type littermates. These data indicate that cisplatin-induced kidney cell apoptosis is associated with impaired IDH2 function and *Idh2* gene deletion aggravates kidney apoptosis.

Taken together, our data demonstrate that cisplatin impairs the mitochondrial IDH2-NADPH-GSH antioxidant system, leading to mitochondrial oxidative stress and consequently, renal cell apoptosis, dysfunction, and damage. This suggests that the mitochondrial IDH2-NADPH-GSH antioxidant system is a useful target axis to prevent cisplatin nephrotoxicity.

## Materials and methods

### Animal experiments

All experiments were conducted using 12-week-old female *Idh2*-gene-deficient (*Idh2*^*‒/‒*^) mice and wild-type (*Idh2*^+/+^) littermates^37^. The study was approved by the Institutional Animal Care and Use Committee of Kyungpook National University. Mice were allowed free access to water and standard mouse chow. Cisplatin (*Cis*-diamminedichloroplatinum II, 20 mg/kg body weight; Sigma, St. Louis, MO, USA) was administered to the mice once intraperitoneally. Some mice were injected intraperitoneally with (2-(2,2,6,6-Tetramethylpiperidin-1-oxyl-4-ylamino)-2-oxoethyl) triphenylphosphonium chloride (Mito-T, 0.7 mg/kg body weight; Sigma) once daily, beginning 7 days before cisplatin injection and continuing until experiments were completed. Mice were killed 3 days after cisplatin injection. Kidneys were either snap-frozen in liquid nitrogen for biochemical analysis or fixed in PLP (4% paraformaldehyde, 75 mM l-lysine, 10 mM sodium periodate) for histological studies.

### Renal functional parameters

Blood was taken from the retro-orbital venous plexus using a heparinized capillary glass tube. BUN and PCr were measured using a Vitros 250 Chemistry Analyzer (Johnson & Johnson, New Brunswick, NJ, USA).

### Histology

PLP-fixed kidneys were embedded in paraffin and cut into 3-μm-thick sections using a microtome (RM2165; Leica, Wetzlar, Germany). Kidney sections were stained with periodic acid Schiff stain (PAS) according to the manufacturer’s instructions. Images were captured using the i-Solution software (IMT, Vancouver, Canada). Tubule damage was scored by the following criteria: 0, no damage; 1, mild damage with rounded epithelial cells and dilated tubular lumen; 2, moderate damage with flattened epithelial cells, dilated lumen, and congestion of the lumen; and 3, severe damage with flat epithelial cells lacking nuclear staining and congestion of the lumen. More than ten fields per kidney section were analyzed.

### Mitochondria isolation from kidney tissue

Mitochondrial and cytosolic fractions were prepared as described previously^38^. Briefly, kidney samples were homogenized thrice in sucrose buffer (0.2 M sucrose, 1 mM EGTA, 10 mM HEPES, pH 7.4). A Teflon Homogenizer (Daihan Scientific, Seoul, Korea) was used at 1600 r.p.m. and this step was performed in an ice bath. The homogenate was centrifuged at 600 × *g* for 10 min at 4 °C. The supernatant was centrifuged at 7000 × *g* for 10 min. To obtain the purified cytosolic fraction, the supernatant was centrifuged again at 7000 × *g* for 10 min. The pellet was washed twice and centrifuged again at 7000 × *g* for 10 min. It was then suspended in sonication buffer (0.1% Triton X-100 in PBS) and sonicated twice using a 4710 series sonicator (Cole-Palmer, Chicago, IL) at 40% maximum setting for 10 s. Subsequently, after centrifugation at 15,000 × *g* for 30 min, the supernatant containing mitochondria was collected. Fractions were confirmed by western blot analysis using anti-COX IV (Abcam, Cambridge, MA, USA) as a marker of mitochondria and anti-GAPDH (NOVUS, Littleton, CO, USA) as a marker of cytosol.

### Measurement of hydrogen peroxide levels in the kidney

Hydrogen peroxide levels were measured in kidney homogenates using xylenol orange (Sigma), a ferric-sensitive dye^39^. Measurement was based on the following principles: H_2_O_2_ oxidizes iron (II) to iron (III) in the presence of sorbitol, which acts as a catalyst; and iron (III) makes a purple complex with xylenol orange.

### Western blot analysis

Western blotting was performed using anti-4-hydroxynonenal (4-HNE; Abcam), anti-peroxiredoxin (Prx-SO_3_, Abcam), anti-MnSOD (Calbiochem, San Diego, CA), anti-copper-zinc superoxide dismutase (CuZnSOD; Chemicon, Temecula, CA), anti-histone H1 (Santa Cruz Biotechnology, Santa Cruz, CA, USA), anti-mitochondrial NADP^+^-dependent isocitrate dehydrogenase (IDH2)^40^, anti-Opa1 (BD Bioscience, Franklin Lakes, NJ, USA), anti-Drp1 (Cell Signaling Technology, Danvers, MA, USA), anti-Bax (5B7; Santa Cruz), anti-Bcl-2 (Cell Signaling Technology), anti-cytochrome *c* (BD Bioscience), anti-cleaved caspase-3 (Merck Millipore, Darmstadt, Germany), anti-β-actin (Sigma), and anti-GAPDH (Novus, Littleton, CO, USA) antibodies.

### Measurement of IDH1, IDH2, and IDH3 activity

The determinations of IDH1 and IDH2, and IDH3 activity were based on the productions of NADPH and NADH, respectively^41^. Briefly, cytosolic fraction for IDH1 activity and mitochondrial fraction for IDH2 activity were incubated in the reaction buffers containing 40 mM Tris (pH 7.4), 100 mM NADP^+^, 100 mM MgCl_2_, and 200 mM isocitrate (Sigma), respectively. Their activities were defined as the changes in absorbance at 340 nm over 1 min at 37 °C. For the determination of IDH3 activity, mitochondrial fraction was incubated in the reaction buffer containing 40 mM Tris (pH 7.4), 100 mM NAD^+^, 100 mM MgCl_2_, and 200 mM isocitrate. Activity was defined as the change in absorbance at 340 nm over 1 min at 37 °C.

### Measurement of mitochondrial NADPH concentration

Measurement of NADPH concentration was based on a glucose dehydrogenase cycling reaction. The level of NADPH in kidney mitochondria was determined using an EnzyChrom™ NADP/NADPH Assay Kit (BioAssay Systems, Hayward, CA, USA) according to the manufacturer’s instruction. In brief, freshly isolated kidney mitochondria was suspended in 100 μL NADPH extraction buffer for NADP determination or 100 μL NADPH extraction buffer for NADPH determination, and then heated at 60 °C for 5 min. After heating 20 μL assay buffer and 100 μL of the NADP, opposite extraction buffer was added to neutralize the extracts. After centrifugation of the sample at 14,000 r.p.m. for 5 min, the supernatant was used for determination. In 96-well plate, 40 μL samples were transferred and added 80 μL working reagent (60 μL assay buffer, 1 μL enzyme mix, 10 μL glucose, and 14 μL MTT). The plate was briefly tapped to mix and read the optical density (OD) at 565 nm at time zero and 30 min. The concentration of NADP(H) was calculated using the change of OD for 30 min.

### Measurement of GSSG and total glutathione (tGSH) levels in the kidney

Measurement of oxidized glutathione (GSSG) and total glutathione (tGSH) are based on enzymatic recycling method for quantification of glutathione; GR reduces oxidized glutathione (GSSG) to reduced glutathione (GSH). The sulfhydryl group of GSH reacts with DTNB (5,5′-dithiobis-2-nitrobenzoic acid) to produce a yellow-colored 5-thio-2-nitrobenzoic acid (TNB) that absorbs at 405 nm. The rate of TNB production is directly proportional to the concentration of glutathione in the sample. The ratio of GSSG to tGSH was measured using the glutathione (GSSG/GSH) detection kit (Enzo Life Sciences, Farmingdale, New York, USA) according to the manufacturer’s instruction. In brief, freshly isolated mitochondrial fractions were suspended in ice-cold 5% metaphosphoric acid (20 μL/mg tissue) and centrifuged at 12,000 × *g* for 15 min at 4 °C. Supernatants reacted to the freshly prepared reaction mix. The absorbances were detected at 405 nm every minute for 10 min. Determination of GSSG was the same protocol with GSH assay with an exception in which mitochondrial fraction was suspended in 5% metaphosphoric acid containing 2 M 4-vinylpyridine.

### Measurement of glutathione peroxidase (GSH-Px) and glutathione reductase (GR) activity in kidney mitochondria

GSH-Px activity was measured in a reaction mixture, including 0.1 M potassium phosphate buffer (pH 7.0) containing 1 mM EDTA, 0.25 U GR, 10 mM GSH, and 1.5 mM NADPH. Measurement of GR activity was performed in the same way, using 0.1 M potassium phosphate buffer (pH 7.0) containing 1 mM EDTA, 10 mM GSSG, and 10 mM NADPH. Activities were measured as the change in absorbance at 340 nm in 1 min at 37 °C.

### Immunofluorescence

Paraffin-embedded kidney sections were deparaffinized, rehydrated, and washed with distilled water. Sections were then incubated with PBS containing 0.2% Triton X-100 for 5 min and washed with PBS for 10 min. For antigen retrieval, kidney sections were heated in 0.01 M sodium citrate buffer (pH 6.0) for 10 min using an autoclave. Sections were then cooled and washed thrice with PBS. Sections were blocked with PBS containing 1% bovine serum albumin for 30 min. Kidney sections were incubated with an anti-8-hydroxy-2′-deoxyguanosine (8-OHdG, Abcam) antibody in a humidified chamber at 4 °C overnight. After three washes, sections were incubated with FITC-conjugated secondary antibodies for 1 h at room temperature and then washed with PBS in the dark. Sections were mounted with the Prolong Gold anti-fade reagent (Invitrogen, Carlsbad, CA, USA). Images were captured using a Leica DM2500 microscope.

### Transmission electron microscopy

Three days after cisplatin or 0.9% saline (vehicle) administration, kidneys were fixed with 2.5% glutaraldehyde at 4 °C for 12 h and cut into 1 mm^3^-sized blocks. Kidney samples were washed in 0.1 M phosphate buffer and post fixed in aqueous 2% osmium tetroxide for 90 min. Samples were then washed thrice with phosphate buffer and dehydrated through a graded series of 50–100% ethanol and 100% propylene oxide and then infiltrated in 1:1, 1:2, and 1:3 mixtures of propylene oxide: Epon Resin 828 (Polysciences Inc., Warrington, PA, USA) for 1 h, respectively. Samples were then incubated with 100% Epon Resin 828 for 8 h, embedded in molds. Samples were cured at 35 and 45 °C for 12 h, with additional hardening at 60 °C for 2 days. Samples were cut into ultrathin (60 nm) sections and double-stained with 2% uranyl acetate and 1% lead citrate. Electron micrographs of mitochondria were obtained from proximal tubular cells in the cortex using a transmission electron microscope (H-7000, Hitachi, Japan) at 75 kV.

### Terminal deoxynucleotidyl transferase dUTP nick end labeling (TUNEL) assay

TUNEL assays were performed using an in situ cell death detection kit (Roche, Basel, Switzerland) according to the manufacturer’s instructions. In brief, kidney sections were incubated with the TUNEL reagent mixture at room temperature for 30 min and washed thrice with PBS for 5 min each time. Images were captured using a microscope (Leica DM2500). TUNEL-positive cells were counted with i-Solution software (IMT, Cicero, NY, USA).

### Statistical analysis

Statistical differences among groups were evaluated by Student’s *t*test. Results were expressed as the means ± SE. Differences were considered statistically significant at *P* values <0.05.
